# The Homœopathic Treatment of Rheumatism and Kindred Diseases

**Published:** 1888-03

**Authors:** 


THE HOMŒOPATHIC TREATMENT OF RHEUMATISM AND KINDRED DISEASES. By D. C. Perkins, M. D. Pp. 180; price, $1.50. Hahnemann Publishing House, Philadelphia. 1888.

It is seldom that we can say, with truth, that a long-felt want is at last supplied, but in reference to Dr. Perkins's book we do say it. There has been no monograph in our school on rheumatism. One has long been needed. This volume gives the homœopathic indications for about one hundred remedies; under each remedy the symptoms of the back and the extremities, with concomitants, are given. At the end of the book is a very good repertory. Rheumatism is often a tough customer to deal with, hence the help of such a work as this will be very welcome.

